# Comparative evaluation of antimicrobial, antibiofilm, antioxidant, antiviral, and antidiabetic activities of copper oxide nanoparticles biofabricated via *Opuntia ficus indica*

**DOI:** 10.1038/s41598-025-08878-3

**Published:** 2025-07-10

**Authors:** Mohamed K. Y. Soliman, Salem S. Salem

**Affiliations:** Botany and Microbiology Department, Faculty of Science, Al-Azhar University, Nasr City Cairo, Egypt

**Keywords:** Nanotechnology, Copper oxide nanoparticles, Green synthesis, Medical application, Nanofabrication and nanopatterning, Nanoparticles, Biomedical materials

## Abstract

The eco-friendly methods of synthesizing nanoparticles using plant extracts have garnered heightened attention. In recent years, copper oxide nanoparticles (CuO-NPs), have found utility in a variety of applications. This work reports the fabrication of CuO-NPs using watery extract of *Opuntia ficus indica* leaves as a stabilizing component. Different analyses were performed to characterize the produced CuO-NPs. The CuO-NPs produced were very stable, spherical, and about 65 nm in size. The antimicrobial potency of produced CuO-NPs was assessed against four Gram-positive and four Gram-negative pathogenic bacteria, as well as uniـcellular fungi, revealing inhibition-zones ranging from 18 to 24 mm and a minimum inhibitory concentration (MIC) between 62.5 and 500 µg/mL. The DPPH was utilised to investigate the free-radical scavenging ability of CuO-NPs at various concentrations, revealing a notable antioxidant capacity with an IC_50_ of 165.5 µg/ml. Moreover, CuO-NPs exhibited various antibiofilm activity versus *S. aureus* (MRSA) and *P. aeruginosa* inhibiting up to 59.3% and 89.4% at 200 µg/mL respectively. A molecular docking simulation revealed that CuO-NPs’ activity against bacterial strains may be due to beta-1,3-glucanase, with hydrophobic interactions with amino-acid residues in the active site. Moreover, CuO-NPs at a concentration of 125 µg/mL had a significant antiviral effect versus HAV and COXB4, with antiviral efficacy of 28.6% and 40.9%, respectively. Furthermore, the CuO-NPs at 1000 µg/mL exhibited 91.5% inhibition of α-amylase and 82.3% inhibition of α-glucosidase, therefore affirming their antidiabetic properties. Consequently, CuO-NPs have significant promise as an anti-inflammatory drug.

## Introduction

One of the most important modern sciences that are applied to many different technologies is nanotechnology, whose influence has been more noticeable recently. This field has garnered more interest in recent years from a variety of scientific disciplines within the research community^1,2^. Metals and metal oxides are among the materials that can produce nanoparticles (NPs). Due to their particular properties, metal and metal oxide NPs bear substantial relevance and show potential for applications spanning from basic research to industrial development^3,4^. Numerous industries, including food processing, agriculture, nanomedicine, and more, have used nanomaterials^5–8^. An inventive nanotechnological approach that has drawn more attention is the incorporation of living cells and bioresources in the creation of NPs. Numerous methods, as chemical, physical method, and biological ones, are used to create metal nanoparticles^9–11^. However, NPs mediated by plant extracts have been found to be safer for medicinal purposes, less poisonous, and more biocompatible than those made by physicochemical means^12,13^. The biological approach, also referred to as “green synthesis,” gained popularity because to its many benefits, including low toxicity of the resultant NPs, cost-effectiveness, safety considerations, removal of hazardous chemicals, and decrease of byproduct waste^14^. Therefore, a viable substitute for chemical and physical method is the biological creation of NPs using plant-cell extracts, which act as both capping and reducing agents^15^. The physical characteristics of nanomaterials as shape and particle diameter affected by the external reaction conditions as pH and temperature^16,17^. However, the synthesis of nanomaterials for certain applications mostly faces numerous limitations, as pronounced instability, potential toxicity, and insufficient understanding of the reaction mechanisms and behavior of the synthesized nanomaterials. While plant-mediated synthesis offers a straightforward approach, microbial synthesis requires additional steps, which can hinder NP production efficiency^18^.

Using NPs to increase the bioavailability of active herbal components is a practical approach. Alkaloids, flavonoids, phenols, tannins, ascorbic acids, and other secondary metabolites are among the phytochemical components of plant leaves that are essential for the creation of metal and metal oxide-based nanomaterials^19^. These phytocompounds help produce metal nanoparticles (MNPs) by stabilizing and reducing metal salts, which ultimately produces NPs with improved stability and biocompatibility. Additionally, these NPs have antibacterial and antioxidant properties^20,21^. Natural products and metal nanoparticles have been shown to have therapeutic benefits in the treatment of a range of infectious and metabolic conditions, such as those associated with inflammation, bacterial infection, and oxidative stress^22^. Numerous research have demonstrated the antibacterial capabilities of CuO-NPs, demonstrating that their efficacy varies according to synthesis techniques, surface charge, and size^23,24^. Bacterial and fungal cell death can result from interactions between CuO-NPs and microbial cell membranes that cause structural damage. Additionally, their compact size enhances their antibacterial effect by allowing for higher cellular uptake. When CuO-NPs are mixed with other natural substances, increased activity is frequently seen, indicating a synergistic effect that may be used therapeutically^25^. Traditional cancer treatments frequently come with negative side effects, pharmacokinetic difficulties, and diagnostic constraints. Nanotechnology offers a promising answer to these challenges. Nanoparticles, characterized by their high surface charge, dimensions, and shapes, have transformed cancer diagnosis and therapeutic approaches^26,27^. Significantly, metal nanoparticles like CuO-NPs have attracted interest because of their advantageous safety profile and lower toxicity^28^. The use of nanoparticles has led to remarkable progress in drug delivery, resulting in extremely precise systems that can target and deliver therapeutic agents to specific cancer cells.

This study aimed to synthesize CuO-NPs quickly and simply via the bioreduction process using *Opuntia ficus indica* leaves extract. Furthermore, the characteristics of these CuO-NPs were examined, along with their potential in medical applications with docking study related to antimicrobial, antibiofilm, antioxidant, antiviral, anti-inflammatory and antidiabetic activities.

## Materials and methods

### Chemicals

Copper acetate (Cu(CH_3_COO)_2_), Dimethyl sulfoxide (DMSO), DPPH (2,2-diphenyl-1-picrylhydrazyl), Muller Hinton agar and broth medium, Potatoes Dextrose broth medium, MTT (3-(4,5-Dimethylthiazol-2-yl)-2,5-Diphenyltetrazolium Bromide), Phosphate-buffered saline (PBS), Dulbecco’s Modified Eagle Medium (DMEM), 3,5-dinitrosalicylic acid (DNSA), pNPG )chromogenic substrate)and sodium-carbonate (Na_2_CO_3_) were procured from Sigma Aldrich (St. Louis, MO, USA).

### Preparation of *Opuntia ficus indica* leaf extract

As the previous study with some alteration^29^, the plant leaves were collected from the garden of the Faculty of Science at Al-Azhar university, followed by established protocol, and permission was obtained. Further, the plant material was identified at Department of Botany and Microbiology, Faculty of Science, Al-Azhar University, Egypt. It was deposited in the department’s herbarium. The plant leaves were washed three times by D.H_2_O then dried in air. The dried leaves of *Opuntia ficus indica* had been crushed with a mortar and pestle. After more crushed fluids were moved, they underwent a 10 min. centrifugation operation at 10,000 rpm to eliminate debris. Boiling the mixture for 45 min at 60 °C, this solution was collected and put in a 250 mL beaker along with 100 mL of purified water. After filtration via filter paper several times, the resultant solution was kept for twenty-four h at room temperature.

### Green synthesis of CuO-NPs 

Next, whilst stirring at 60 °C, we mixed 45 mL of 0.1 M copper acetate to 100 mL of *Opuntia ficus-indica* produced water extract. We developed green manufactured CuO-NPs, which were centrifuged for 10 min. at 5000 rpm to eliminate the impurities. The obtained pellet had been heated at 200 °C for 3 h^30^. This sample was stored at room temperature for additional studies.

### Characterization of CuO-NPs

CuO-NPs were characterised at a spatial resolution of 1 nm and also in the 200–800 nm wavelength spectrum employing the Tokyo, Japan, JASCOـV 560 UV–vis spectroـphotometer. Prior to estimating them, the samples had been diluted ten times with deionized water to investigate particle size. TEM microscopy using the JEOL JEM-100 CX model (Peabody, MA, USA) was utilized to assess the size and shape of the produced CuO-NPs. Drop coating the CuO-NPs on carbon-coated TEM layers allowed for TEM imaging to be performed. SEM coupled to a JEOL JSM ـ6510 LV energy dispersive spectroscopy (EDS) instrument was used to examine the surface morphology as well as elemental compositions of CuO-NPs. The size of the particles of CuO-NPs distributed in water was measured using dynamic-light scattering (DLS). FTIR employing the potassium bromide (KBr) technique was used to identify the functional groups present in the biomass of bacterium filtrate and how they are used in the production of CuO-NPs. A sample of CuO-NPs powder was combined with KBr for this study, and the mixture was thoroughly compressed to create a disk. The disk was then scanned within 400–4,000 cm^−1^. The rectified sample was centrifuged, and the precipitate was removed for XRD examination after being vacuum-dried. XRD-6000 series was used to produce X-ray diffraction patterns, which were then superimposed to analyze materials and perform stress-analysis, residual austenite quantification, NPs crystallite size, and crystallinity computation. The Shimadzu apparatus, manufactured by Shimadzu-Scientific Instruments (SSI) in Kyoto, Japan, uses a nickel-filter and a Cu-Ka target. The Debye ـScherrer equation, D= kλ/β Cos θ, was additionally employed to calculate the median crystalline size of the CuO-NPs. In this instance, λ is the X-ray wavelength, β is the full width at half maximum, D is the mean crystalline size (nm), k is the Scherrer constant (0.9–1), and θ is the Bragg diffraction angle (degrees). These estimates comprised materials evaluation using superimposed X-ray diffraction models, stress research, residual austenite quantitation, crystallite capacity, and crystallization consideration^5,28^.

### Antimicrobial efficacy

#### Agar well diffusion technique

CuO-NPs were evaluated versus nine different microbial strains (*Staphylococcus aureus* (MRSA &MSSA), *Proteus vulgaris*,* Bacillus subtilis*,* Streptococcus pneumoniae*,* E. faecalis*,* E. coli*,* K. pneumoniae* and *C. albicans*) CuO-NPs were evaluated for their antimicrobial activity utilizing the agar well diffusion technique^31^. The bacterium suspension of 1.5 × 108 cfu/mL was achieved by adjusting the microbial-cultures to a standard concentration of 0.5 McFarland. Employing the spread plate approach, Petri plates received inoculation with the chosen microbes to supply every trial after being filled approximately 25 mL of Muller ـHinton agar medium. Following a 30 min, the 6 mm-diameter wells were properly cut into the agar plates and subsequently loaded with 100 µL of dissolved CuO-NPs in DMSO. All the plates were then incubated for 24 and 72 h at 37 °C and 28 °C. Three duplicates of each experiment were conducted to guarantee accuracy. To reduce mistakes and provide trustworthy findings on CuO-NPs’ antimicrobial activity versus the investigated microbial strains, this technique was used. Using the micro-well diluted test, the MICs of CuO-NPs were ascertained towards all microbial strains. After the turbidity of the Muller Hinton broth and Potatoes Dextrose broth (OXOID Ltd., Basingstoke, UK) equaled 0.5 McFarland, the microbial specimens were added. Ten microliters of the suspensions of microbes and ninety microliters of broth media were added to each well of 96-well sterile microplates to conduct the test. Next, 100 µL of CuO-NPs, with concentrations varying (0.8, 0.7, 0.6, 0.5, 0.4, 0.3, 0.2, 0.1 and 0.05 mg/mL), were introduced into the wells. The microbial plates were incubated at 37 °C and 28 °C for a 24 and 72 h. Using an ELISA microtiter plate reader from Thermo FisherـScientific, Inc. in the Chinese city of Shanghai, the optical density (O.D.) has been determined at 595 nm. The MIC was found to be the lowest concentration of CuO-NPs that prevented microbes from growing visibly. Every experiment was run three times^32^.

#### Bioflm inhibition assay

Based to the previous work, the MTP technique was utilized to assess CuO-NPs’ capacity to prevent or lessen the development of bacterial biofilms versus clinical bacterial biofilm producer isolates *S. aureus* and *P. aeruginosa*^33,34^, with a few changes. In summary, CuO-NPs were disseminated at varying quantities into a flat- MTP that contained trypticـsoy broth medium (TSB) enriched by 1% glucose. Test strains diluted 1:100 in TSB with an inoculum size of 1.5 × 106 CFU/ml were cultured overnight and then placed onto MTP, where they were incubated for 48 h at 37 °C. Planktonic cells were transferred over the dishes following the incubation tim. After that, the well materials were moved without affecting the biofilms that had grown, and the MTP wells subsequently repeatedly cleaned with Phosphate-buffered saline (PBS), which has a pH of 7.4, to get rid of any remaining floating unbounded cell debris. For 10 min, 200 µl of 95% methanol was administered to fix the layers of biofilm that had grown in each well. Using a multichannel micropipette (CAPP), 0.3% w/v of crystal violet was introduced to every well. The plates were then allowed to sit at room temperature for 15 min. Furthermore, the plates underwent a gentle washing with sterile distilledـwater after the excess crystal violet stain was eliminated. At this stage, the biofilm-bound crystal violet was inspected and captured on camera using an Olympus Ck40 × 150 inverted microscope. In order to identify quantitative biofilm development, 30% acetic acid was applied to each well, and an automated microplate reader (Tecan Elx800) was used to quantify the absorbance color at 540 nm. The wells that received treatment and those that did not were contrasted.

#### Molecular docking simulation

The molecular docking simulation process for CuO-NPs was performed using Molecular Operating Environment (MOE) version MOE 2019.0901. The 3D structure of target receptors beta-1,3-glucanase (PDB: 2CYG) was downloaded from the protein data bank. The active site of receptors was generated using only one chain (chain A). Beta-1,3-glucanase (PDB: 2CYG) was constructed with an active site generated by selecting (chain A) and generating the active site according to usual methodology. Triangle matcher placement and London dG and GBVI/WSAdG as Rescoring 1 and 2, respectively, were used to finish the docking process. Forcefield was also utilized for post-placement refining. With a negative value, the docking stance had the most binding energy.

#### Antioxidant assay of CuO-NPs

The 1,1-diphenyl-2-picryl hydrazyl (DPPH) method was used to measure the free radical-fighting capacity of different concentrations of CuO-NPs, hence evaluating their efficacy as antioxidants. A 0.1 mM DPPH in ethanol was produced. A mL of the resultant mixture was combined with 3 mL of varying concentrations of CuO-NPs, ranging from 500 to 7.8 µg/mL, in Ethanol prepared by the dilutionmethod. Following thorough agitation of the chemical-reaction mixture including DPPH and CuO-NPs, allow it to rest at 20 °C for 30 min. The absorbance value at 517 nm was measured. This series of experiments used ascorbic acid as the principal standard reagent. The CuO-NP dosage required to inhibit 50% of the free radical produced by DPPH is referred to as the IC_50_ value, which has been calculated using a logarithmic dose-inhibition curve.

#### Antiviral activity

Distribute 10^3^ cells using 200 mL of water each well in a 96-well plate. To facilitate controls, all of the wells must stay unoccupied. Incubate nightly at 37 °C with 5% CO_2_ to facilitate cell adhesion in the plate wells. Incubate the material under study for an h with an innocuous dosage of power source mixed in a 1: 1 V/v ratio with the virus. Introduce 100 µl then let the viral mixture to incubate for a period. Position it on an unstable and rotate at a velocity of 150 rpm for a duration of 5 min. To initiate the virus’s activity, incubate it for 24 h at 37 °C with 5% CO_2_. Prepare a minimum of 2 ml of the mixture using 5 mg/ml of MTT in PBS for each 96-well plate. Dispense 20 µL of the solution for MTT into every well. To thoroughly incorporate the MTT into the medium, put it on an unstable surface and spin at 150 rpm for five min. Place the MTT reagent at 37 °C in a 5% CO_2_ atmosphere for one to five h to facilitate metabolic activity. If required, eliminate any residues by use tissue paper to absorb the medium from the dry plate. Formazan, an MTT metabolic product, may be reconstituted in 200 µL of DMSO. Combine the formazan and solvent in a shaker, then agitate at 150 rpm for 5 min to achieve complete homogenization. Assess optical density at roughly 560 nm while eliminating interference at 620 nm. A definite link should exist between cell amount and optical density^35^.

### Anti-diabetic assay

#### α-amylase test

Using the 3,5-dinitrosalicylic acid (DNSA) method, the study was conducted. After initially dissolving in 10% dimethyl sulfoxide (DMSO), the CuO-NPs were incorporated to buffer phospate at pH 7.0 to more thoroughly dissolve it, giving levels (1.9 to 1000 µg/mL). After mixing 200 µL of CuO-NPs to a 2 units/mL α-amylase liquid form, the mixture was let to sit at 32 °C for a duration of 9.0 min. Subsequently, 200 µL of the 1% liquefied starch (w/v) solution was added to tubes, and they remained vacant for three min. The reaction that was performed had been heated in a boiling water bath about 10 min at 95 °C afterwards it was stopped with 200 µL of DNSA. After allowing the liquid cool down to 20 °C, 5.0 mL of distilled water was added for diluting it. After that, a UV-Visible MiltonـRoy310 (Tokyo; Japan) spectro-photometer was used to find the wavelength approximately 540 nm^36^.

#### α-glucosidase test

The specimens undergoing study were to have their α-glucosidase activity tested. As stated throughout the α-amylase experiment, 10 µL of the sample comprising a variety of dosages were placed in the 0.1 M phosphate buffer (pH 6.8) and the α-glucosidase enzyme solution (1.0 U/mL) and allowed to sit for 20 min at 35 °C. The chemical process was initiated by injecting 20 µL of 1 M pNPG )chromogenic substrate) after 20 min. The mixture’s ingredients were all left for thirty-five min. The method needed the addition of 50 µL of 0.1 N sodium-carbonate (Na_2_CO_3_), and the MiltonـRoy (Tokyo; Japan) 310 plus spectrophotometer was used to determine the wavelength at 405 nm^37^.

## Results and discussion

### Charcterization

**Fig. 1:**
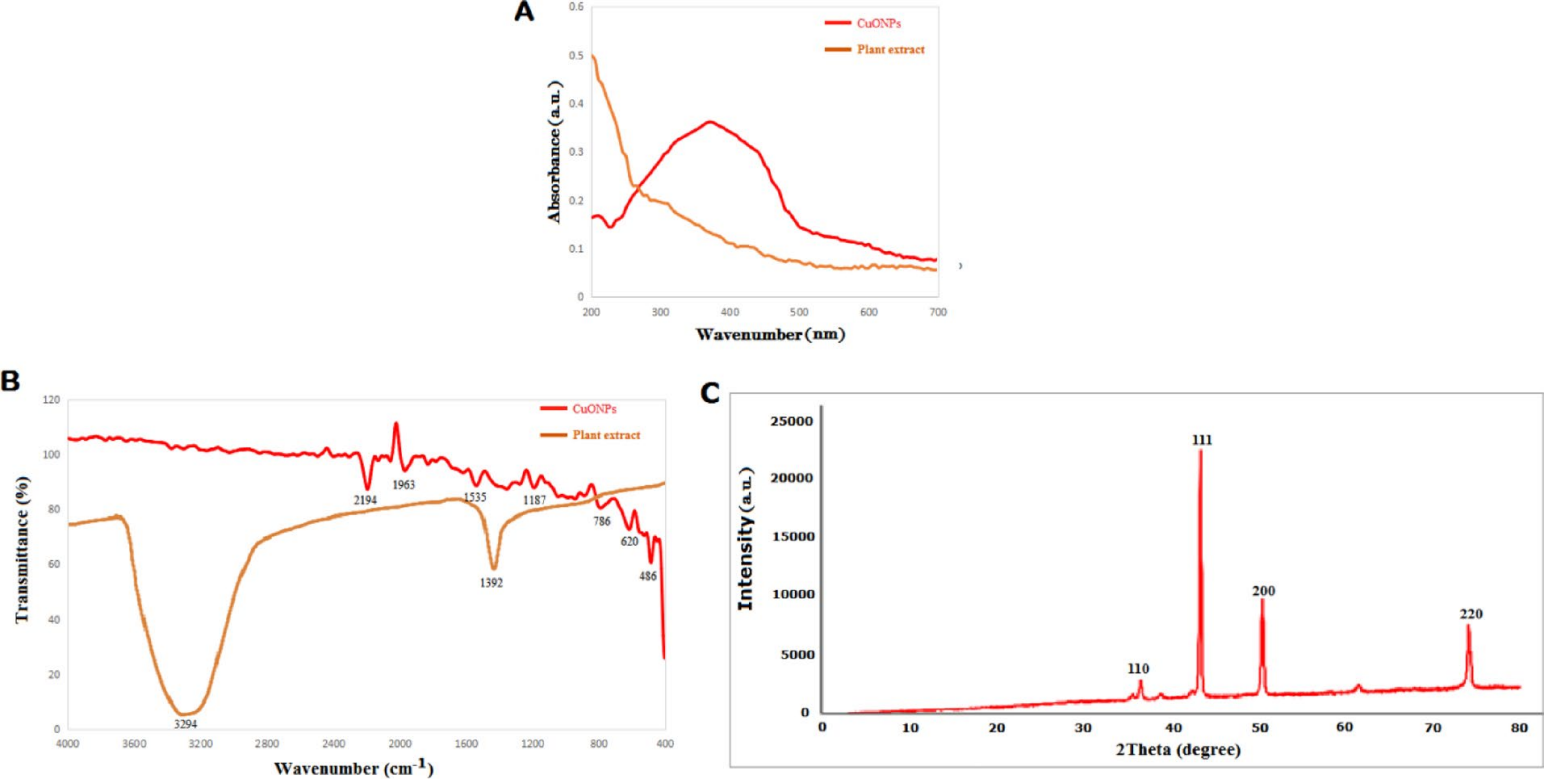
UV–Visible (**A**), FTIR (**B**), and XRD (**C**) spectra of biosynthesized CuO-NPs.

The CuO-NPs’ surface light absorption was examined using UV-Vis. This nanoparticle’s UV range was 200–700 nm, with a maximum absorption around 370 nm (Fig. 1A). It is comparable to CuO-NPs’ UV range. It makes the existence of CuO-NPs known^38,39^. The extract from *Opuntia ficus indica* may function as a capping, reducer, and stabilizer, converting the copper salt into copper oxide nanoparticles. Nonetheless, an absorption peak at around 272 nm was displayed by the biosynthesized nanoparticles made with *E. alata* extract, indicating the successful production and pure phase of CuO-NPs^40,41^. The *Opuntia ficus indica extract* and prepared CuO-NPs’ FTIR spectra revealed a sharp band at 3294, 2194, 1963, 1535, 1392, 1187, 941, 786, 620, and 486 cm^−1^. These bands, respectively, correspond to -O-H bending,-C-O stretching, Amid bonds and -C = C- stretching, Metal-OH (Fig. 1B). The CuO-NPs that were generated had functional groups in them. According to this IR spectrum, flavonoids, proteins, nucleic acids,, phenols, and alkaloids reduce and stabilize those nanoparticles by acting as a capping-agent^30^. The structure of the nanoparticles is preserved under alkaline circumstances by those metabolites as well as chemicals^42^. Green biosynthesised CuO-NPs’ XRD spectra showed a variety of difraction-patterns at 36.35°,43.33°, 50.45°, and 74.1° corresponding to the (110), (111), (200), and (220), respectively (JCPDS, Card code. 65-9743)^43^ (Fig. 1C). The mean particle size of the formed CuO-NPs was determined employing the Scherrer equation, yielding an average crystallite-size of 42.75 nm. The results of the diffraction analysis suggests that the peaks of CuO-NPs exhibit significant good peaks, signifying that the products that are generated are extremely crystalline^43^. The planes found in XRD indicate the creation of a pure monoclinic-structure of CuO-NPs, with no additional peaks detected in the plots^44^. Therefore, it may be proposed which the plant extract significantly influences the size within the bio-reduced CuO-NPs.

**Fig. 2:**
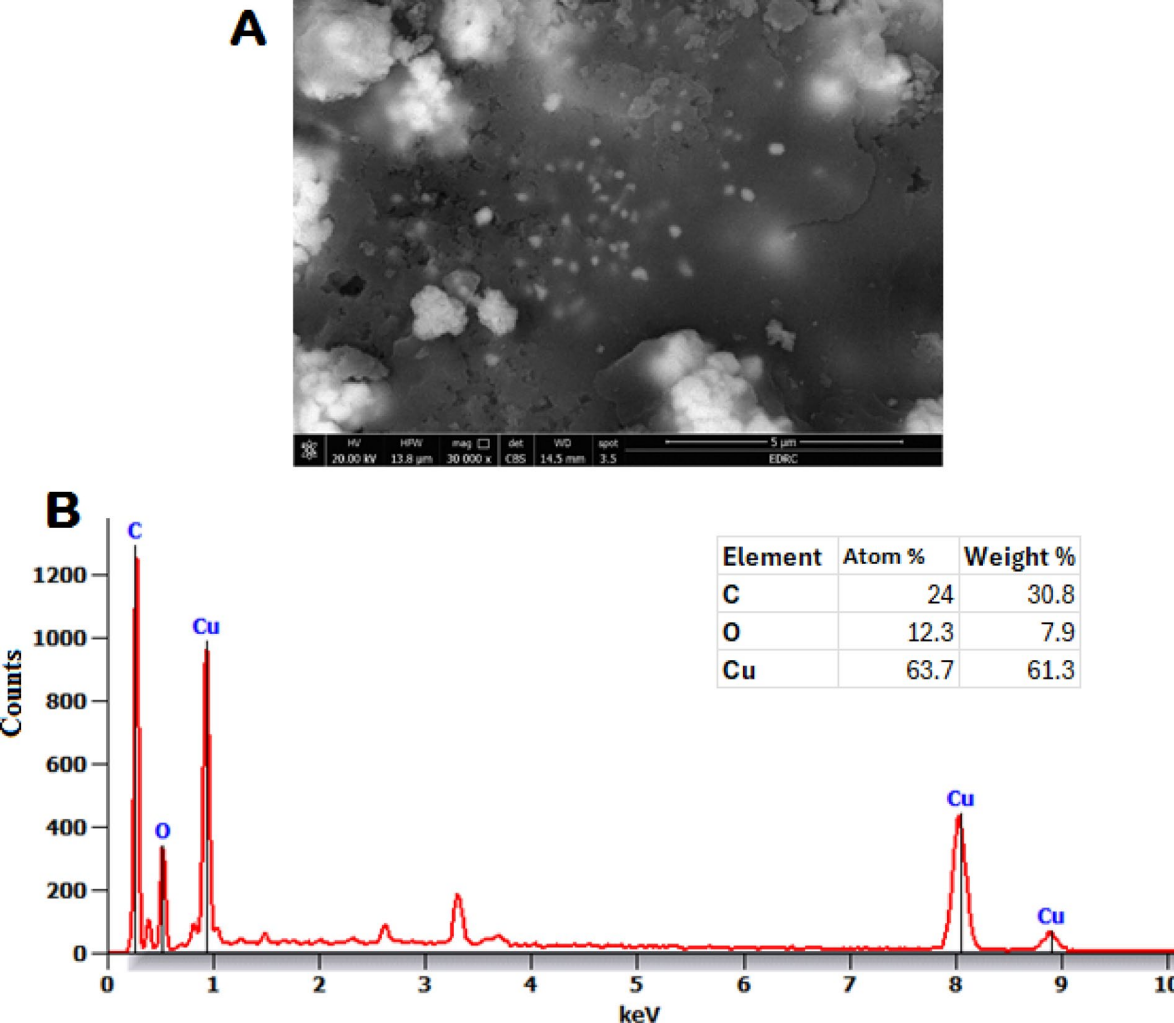
SEM (**A**) And EDX analysis (**A**) of biofabricated CuO-NPs.

The morphology and dimensions of the CuO-NPs have been investigated through SEM, while EDX confirmed the presence of elements inside the CuO-NPs. The leaf extracts of *Opuntia ficus* indica facilitated the formation of CuO-NPs, which exhibit coarse agglomerates and irregular spherical morphologies (Fig. 2A). EDX analysis confirmed that the highest amounts of elemental Cu and O peaks were seen in CuO-NPs (Fig. 2B). These findings validated previous study data about CuO-NPs derived from leaves extract of *Psid. guajava*^45^. The dimension of nanoparticles made of CuO was throughout the acceptable range of previously synthesized dispersion nanoparticles^46^. Furthermore, the nanoparticles were aggregated, maybe attributable their diminutive size, enhanced area of coverage, and diverse molecules^47^.

**Fig. 3:**
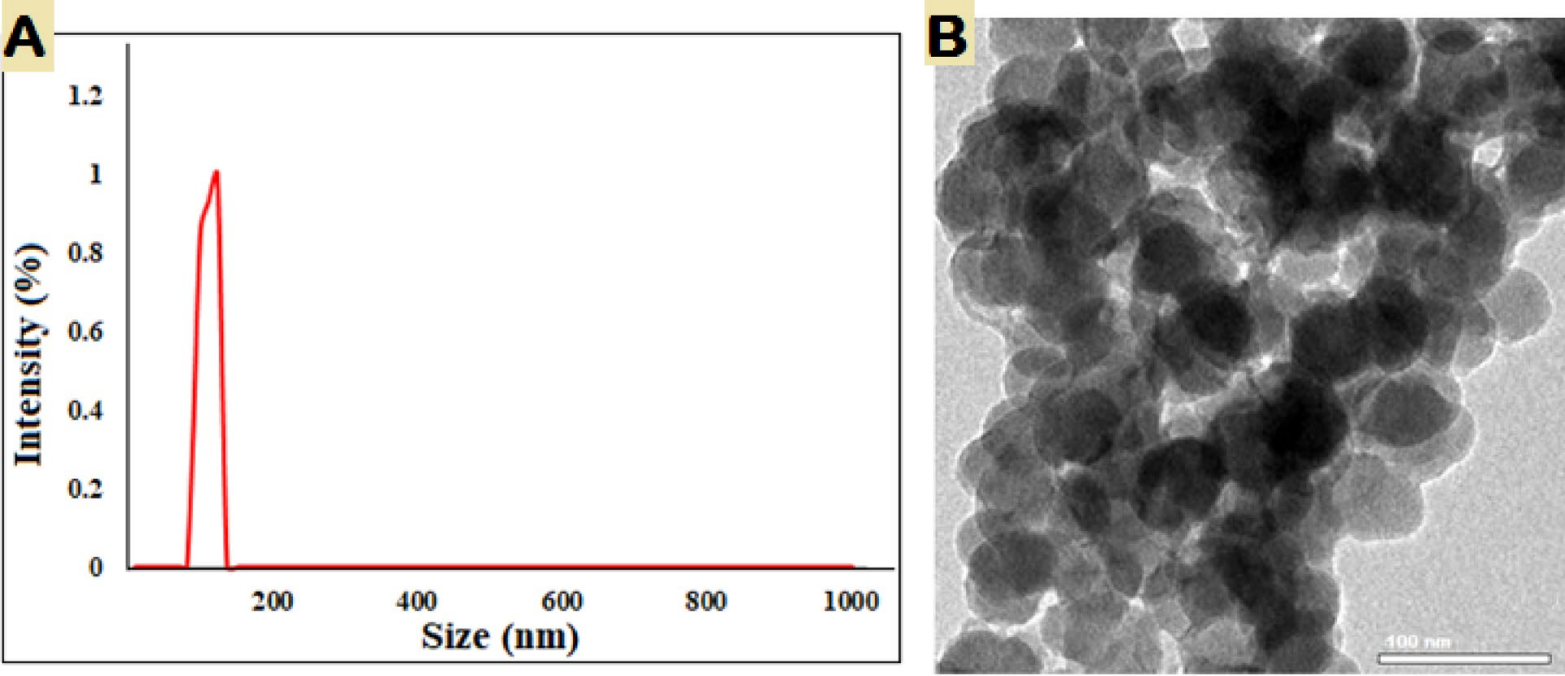
DLS (**A**) and TEM (**B**) of biofabricated CuO-NPs.

The typical particle dimension and surface electrical charge of nanoparticles has been determined through DLS analysis. Figure 3A indicates that the mean particle size variation among green generated nanoparticles composed of copper oxide was 123.3 nm, which is equivalent to the 50 nm size of CuO-NPs produced with *Sida acuta* extract^48^. TEM pictures of produced CuO-NPs are in Fig. 3B. A TEM investigation of phytosynthesize CuO-NPs sized particles as well as shape on the surface showed polydisperse, spherical CuO-NPs with avarage size 65 nm. Padmavathi et al. found that manufactured CuO-NPs are surface components and can reduce CuO to NPs^49^. The solution of sodium hydroxide serves as a catalytic agent, preventing the aggregation of CuO-NPs. The TEM findings of CuO-NPs were entirely congruent with the XRD pattern of the acquired CuO-NPs. This discovery was supported by the findings of Fardood et al., who observed the FCC structure of CuO-NPs by TEM and SAED patterns derived from CuO-NPs generated using *Morinda citrifolia* extract of leaves^50^.

### Antimicrobial activity

**Fig. 4:**
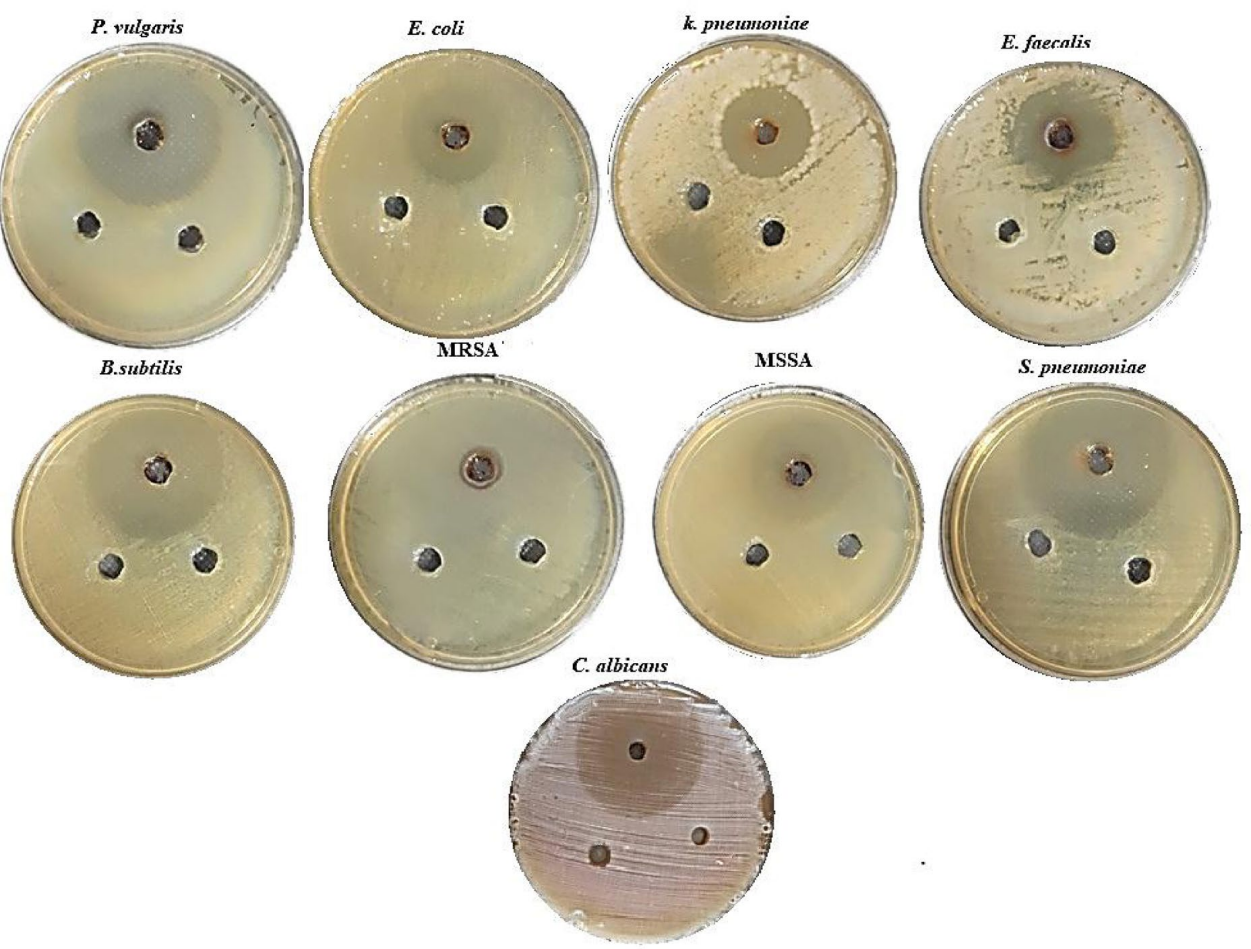
Antimicrobial activity of biofabricated CuO-NPs via agar well.

The antimicrobial activity of CuO-NPs towards several pathogenic microbes was assessed, and the width of the inhibition region within each well is shown in Fig. 4. Specifically, CuO-NPs obtained from *Opuntia ficus indica* leaf extract exhibited a greater zone of inhibition against *P. vulgaris*, *C. albicans* and *B. subtilis*, while demonstrating the least zone of inhibition for MRSA and *E. faecalis*. The results suggest that CuO-NPs obtained from *Opuntia ficus indica* leaf extract are a more promising source of antibacterial chemicals. The findings illustrate the MIC values of phyto-synthesized CuO-NPs. The findings indicate that CuO-NPs synthesized from *Opuntia ficus indica* leaf extract demonstrated concentrations of 0.05, 0.3, 0.4, 0.3, 0.1, 0.8, 0.4, 0.2 and 0.05 mg/mL against *P. vulgaris*,* E. coli*,* K. pneumoniae*,* E. faecalis*,* B. subtilis*, MRSA, MSSA, *S. pneumoniae* and *C. albicans* respectively. CuO-NPs synthesized using *Mangifera indica* fluid extract have shown significant antibacterial efficacy against both *E. coli* and *S. aureus* strains^51^. Furthermore, copper oxide nanoparticles produced from Curcuma longa shown significant-antibacterial efficacy against *P. aeruginosa* and *P. vulgaris*^52^. CuO-NPs synthesized via *Allahabad Safeda* extraction shown enhanced antibacterial activity attributable to their superior chemical reactivity and chemical composition on the surface, facilitating effective contact with microbes^53^. Their little size augments their capacity to engage with bacteria and bolster overall antimicrobial capabilities. CuO-NPs affect the external membrane, resulting in structural alterations, disintegration, and cell lysis^54^. They additionally impede the DNA reproduction of bacteria, leading to detrimental consequences^55^. Furthermore, the charge they carry facilitates electrostatic forces with the conductive membranes of cells of microbes, resulting in their demise. Furthermore, CuO-NPs obtained from herbal extracts generate reactive oxygen species (ROS) that may harm the DNA and cellular membranes of microorganisms, hence impeding their proliferation^54^. Moreover, powerful metabolites derived from CuO-NPs may interfere with bacterial quorum-sensing systems, hence inhibiting their ability to induce outbreaks. CuO-NPs produce copper cations that by electrostatic force adhere to bacterial cell envelopes and permeate the bacteria’s membrane, therefore enhancing their bactericidal efficacy^56^. In overall, CuO-NPs generated from extracts from plants exhibit many antimicrobial techniques, involving chemical interactions with cell membranes, suppression of DNA replication, production of reactive oxygen species, and disruption to microbial quorum sensing. These features render CuO-NPs effective in combating harmful bacteria^57^.

### Antibiofilm

**Fig. 5:**
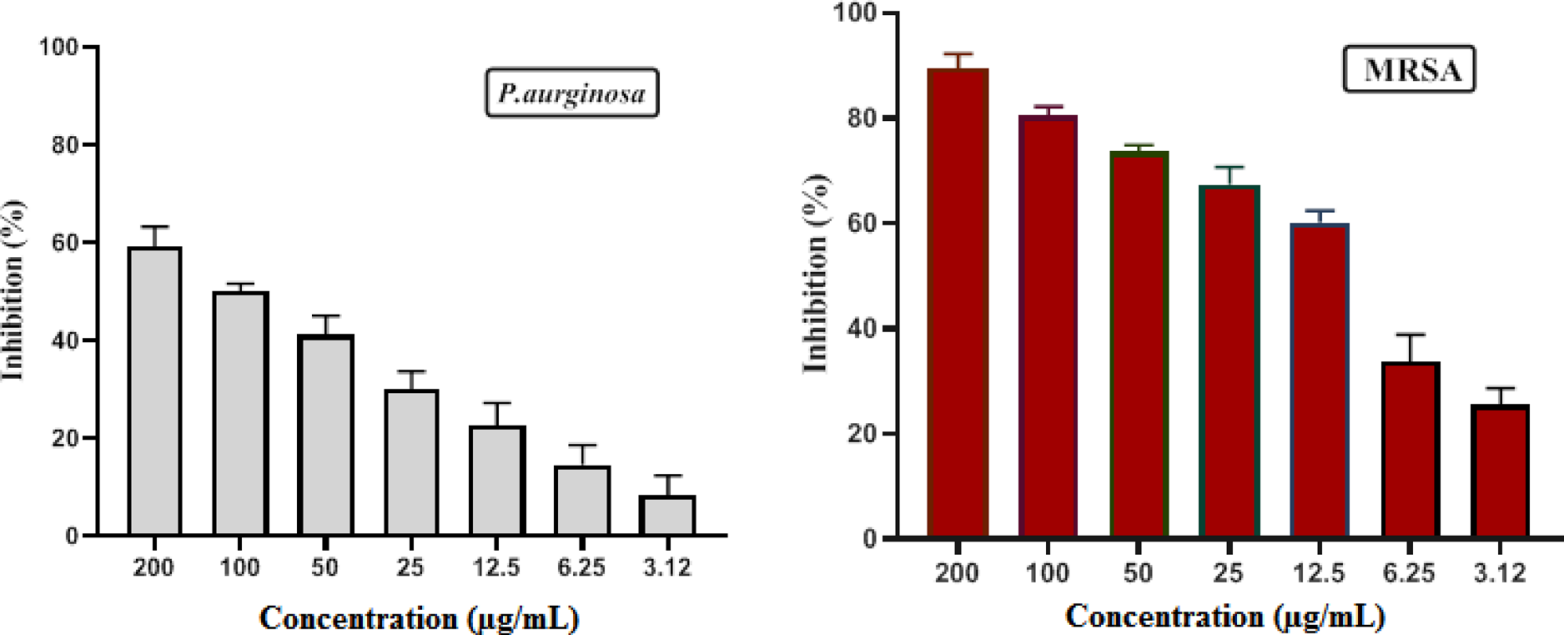
Antibiofilm activity of biofabricated CuO-NPs versus MRSA and *P. auroginosa*.

The antibiofilm impact of copper oxide nanoparticles was assessed by cultivating biofilms with various amounts of nanoparticles, thereafter identifying the adherent cells with crystal violet^34,58^. Figure 5 illustrates a dose-dependent reduction in the biofilm-forming ability of the target bacterium, with an increase in nanoparticle concentration considerably affecting MRSA more than *P. aeruginosa*. CuO-NPs had a slight inhibitory effect versus *P. aeruginosa*, whereas demonstrating the most significant impact on biofilm formation of MRSA, inhibiting up to 59.3% at 200 µg/mL and decreasing to 8.3% at 3.12 µg/mL for *P. aeruginosa*, but for MRSA, the inhibition reached 89.4% and decreased to 25.4% at 3.12 µg/mL. The creation of biofilms is a primary factor contributing to the establishment of drug resistance in pathogenic bacteria. To address infections triggered by biofilm-forming bacteria, it is essential to interfere with or eradicate the biofilm, given that these microorganisms are unresponsive to traditional antibiotics, necessitating the pursuit of new biofilm objectives^59^. Consequently, this work examined not only the antibacterial efficacy of CuO-NPs but also their antibiofilm activity against multidrug-resistant target microorganisms. The findings indicated that CuO-NPs markedly suppressed biofilm development by the target bacteria in a dose-dependent manner. Numerous research have shown the efficacy of CuO-NPs as biofilm inhibitors^60,61^. The outcomes they obtained corroborated the findings of Shehabeldine et al., who similarly demonstrated the antibiofilm efficacy of mycogenic CuO-NPs, seeing 59% and 49% suppression of biofilms versus *K. oxytoca* and *E. coli*, respectively, at subinhibitory doses of nanoparticles^60^. Chari et al. also observed over 60% inhibition of the creation of biofilm in all examined marine pathogens by CuO-NPs manufactured utilizing a one-pot approach^62^.

### Docking simulation

**Fig. 6:**
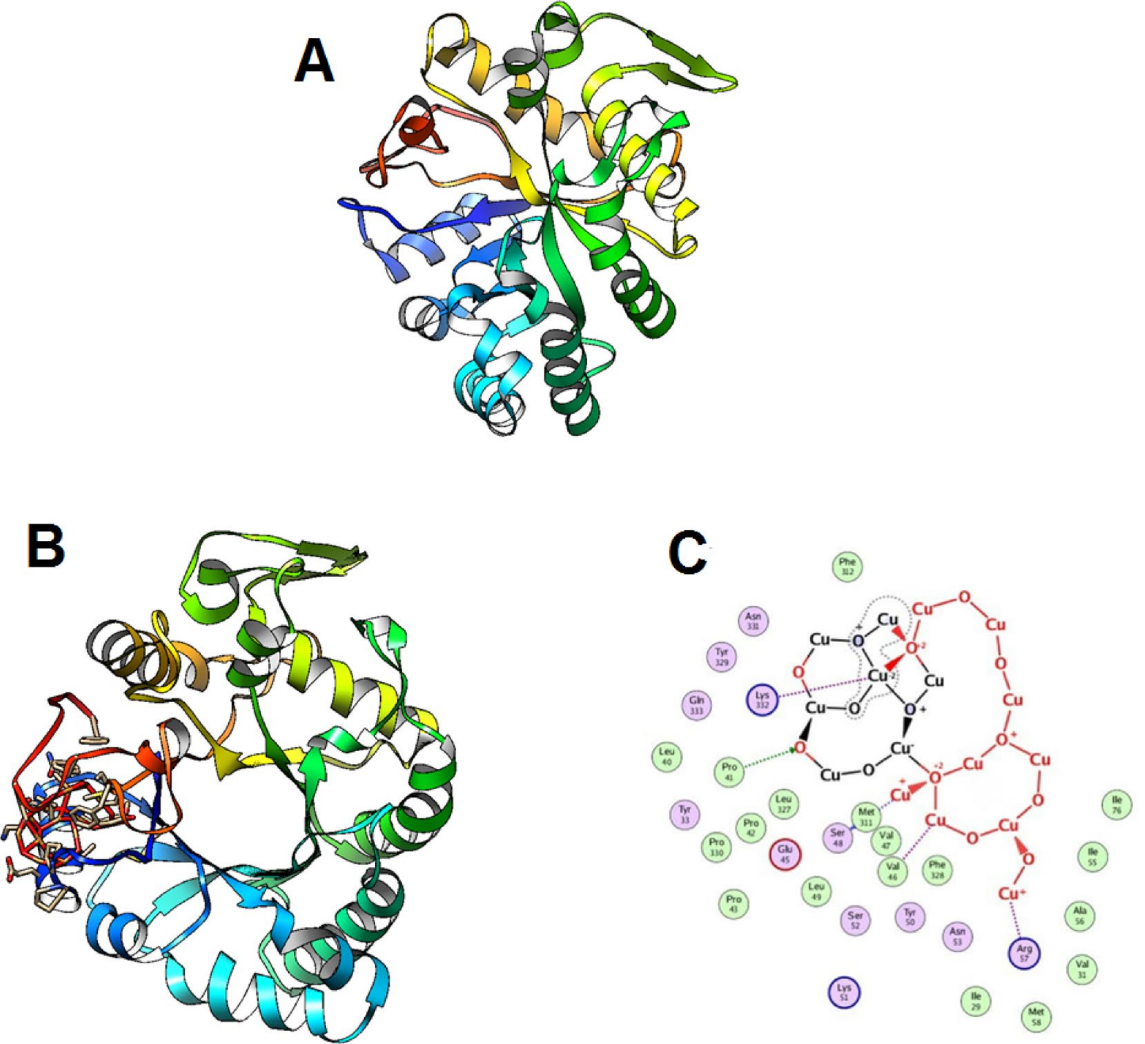
(**A**) 3D crystallography of beta-1,3-glucanase, (**B**) 3D, and (**C**) 2D Structure of the CuO-NPs within the active site of beta-1,3-glucanase (MOE version MOE 2019.0901).

Molecular docking simulation is a crucial method when using computer models to predict the three-dimensional structure of protein-ligand complexes and understand their interactions^63,64^. Furthermore, the nanomaterials docking simulation might shed light on possible action paths and response mechanisms. The CuO-NPs in the beta-1,3-glucanase (PDB: 2CYG) active sites were subjected to docking simulation in this study (Fig. 6A). To find potential targets for antibacterial action, the study set out to ascertain how CuO-NPs interacted with specific enzymes. Initially, the co-crystallized ligand and beta-1,3-glucanase (PDB: 2CYG) root mean square deviation (RMSD) values were acquired. The corresponding values were 0.9412 and 1.17 Å. In the active site of the beta-1,3-glucanase enzyme (PDB: 2CYG), the CuO-NPs demonstrated hydrophobic interactions with numerous amino acid residues, including Pro41,Val46, Ser48, Arg57, and Lys332 (Fig. 6B and C), and a binding energy S = -2.8688 kcal/mol. In the end, we may conclude that CuO-NPs antibacterial action is due to their suppression of beta-1,3-glucanase. However, the binding energy indicates that beta-1,3-glucanase is more preferred by CuO-NPs, and that hydrophobic interactions in the active site pocket, which is located less than 10.8 Å away, indicate their activity.

### Antioxidant activity

**Fig. 7:**
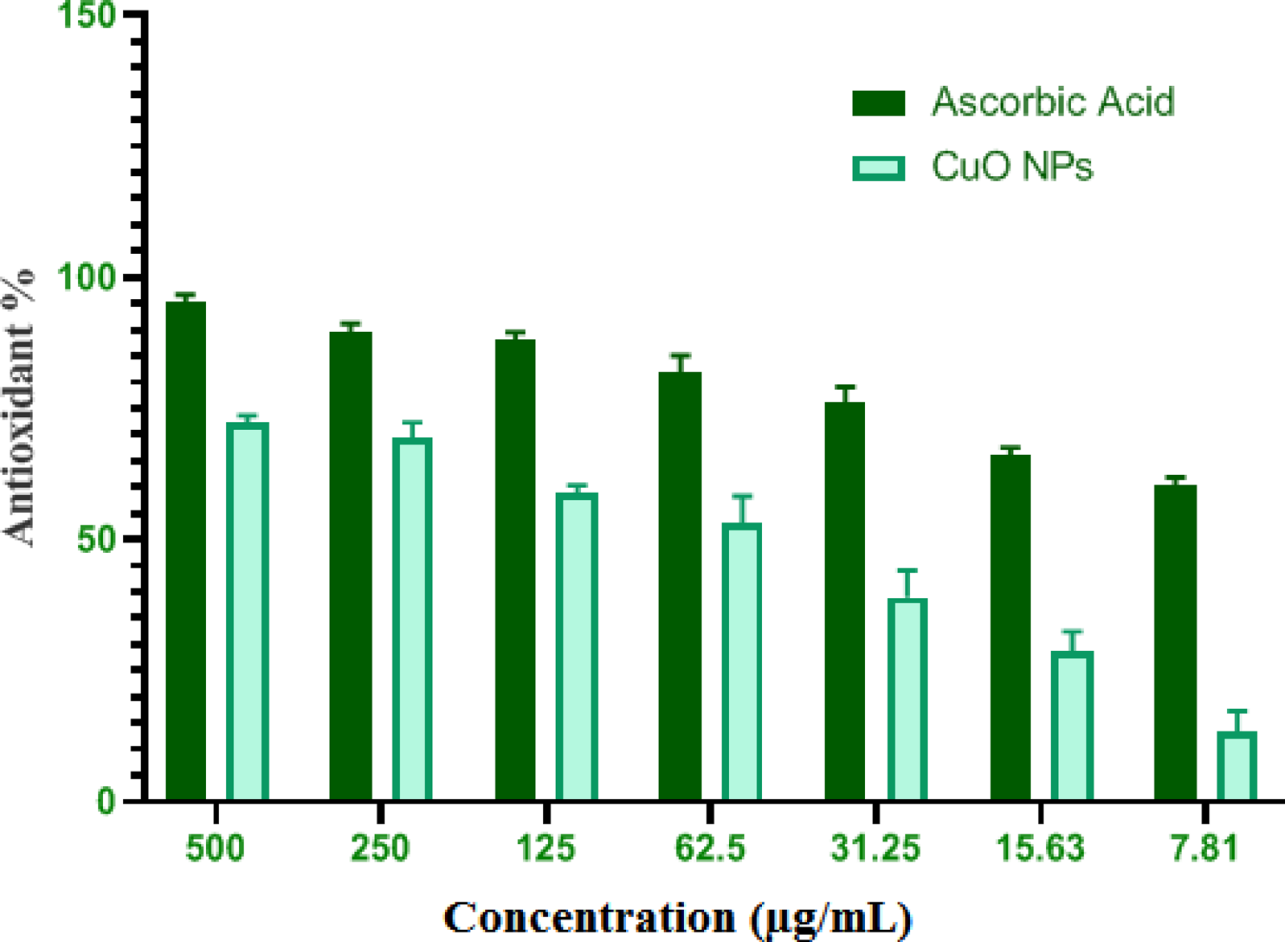
Antioxiant activity of biofabricated CuO-NPs via DPPH.

The contemporary examination of the antioxidant properties of nanoparticles for integration with biological systems is a crucial issue. Free radicals are unstable molecules that are generated in several biological processes as a result of the relationship among molecular oxygen as well as biomolecules^65^. The aforementioned radicals possess a number of unpaired electrons that are marked by high instability, resulting in destruction of biological molecules by extracting electron within them to achieve stability^55^. Different processes, including peroxide breakdown, chain initiation blocking, molecular oxygen abstraction prevention, free radical scavenging, and reductive capacity, have contributed to the antioxidant activity of both synthetic and natural substances^66^. The DPPH scavenge test is the predominant technique for assessing the antioxidant properties of novel active substances. The present work examined the antioxidant capacity of green produced CuO-NPs employing the DPPH scavenging technique. The antioxidant capability of CuO-NPs appeared directly correlated to the quantity of CuO-NPs, as seen in Fig. 7. This finding aligns with the existing literature about the antioxidant properties of green produced CuO-NPs ^55,65^. At an amount of 500 µg mL^−1^, the naturally produced CuO-NPs exhibited a scavenging effectiveness of 72.1 ± 1.4%, whereas ascorbic acid demonstrated a scavenging efficiency of 95.3 ± 1.3% (Fig. 7). The proportions were reduced at minimal concentrations. The scavenge ratios were 58.7 ± 1.6% for CuO-NPs and 88.2 ± 1.3% with ascorbic-acid at a dosage level of 125 µg mL^−1^. The CuO-NPs synthesized using the water-soluble extract of *Suaeda maritima* the heartwood exhibited antioxidant property, as measured by DPPH scavenging techniques, having a value of 83.9% at an amount of 40 µg mL^−1^, in comparison to ascorbic acid, which exhibited 95.2% at the exact same dosage^56^. According the findings of the authors, at the dosage of 5 µg mL^−1^, the scavenging capacity of ascorbic acid along with CuO-NPs dropped according to measured readings of 6.5% and 3.5%, correspondingly. Additionally, at an amount of 300 µg mL^−1^, CuO-NPs made from plant extracts of *Prunus africana* as well as *Camellia sinensis* demonstrated antioxidant effects with proportions of 28.8% along with 28.5%, consequently, in contrast to ascorbic acid’s value of 70.8% at the identical dosage^67^. It was determined the effective amount of CuO-NPs required to scavenge 50% for the free radicals (IC_50_). As demonstrated, the IC_50_ amount for ascorbic acid remained 4.21 µg mL^−1^, whereas that of CuO-NPs was 57.3 µg mL^−1^. Comparable to ascorbic acid, which had an IC_50_ of 23.7 µg mL^−1^, produced CuO-NPs had an IC_50_ of 28.1 µg mL^−1^^56^.

### Anti-diabetic activity of CuO-NPs

**Fig. 8:**
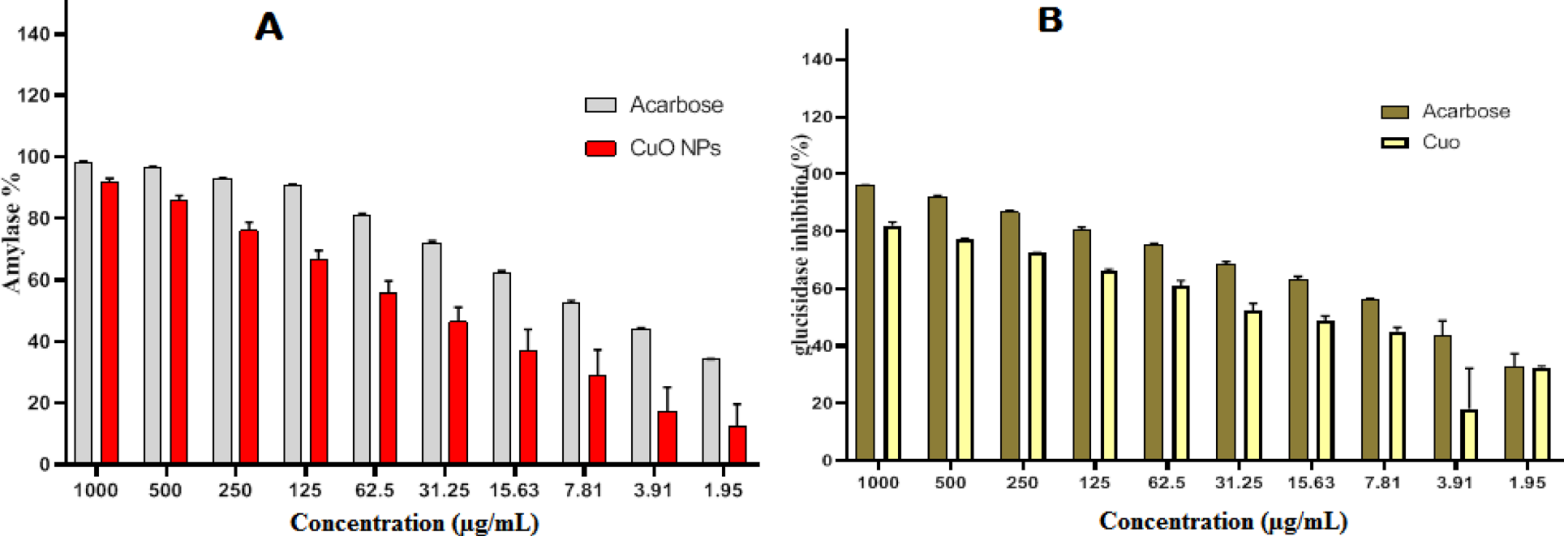
The effect of biofabricated CuO-NPs α-amylase (**A**) & α-glucosidase (**B**) enzymes.

The degradation of α-amylase as well as the gastric enzymes α-glucosidase leads to the formation of di-saccharides and oligo-saccharides^68^. Type-2 diabetes is a medical condition that involves hyperglycemia and polyuria. It is often induced by insufficient insulin levels. It refers to a chronic condition marked by elevated blood glucose and insulin levels. It results from diminished insulin efficacy and the increased hepatic glucose generation^69,70^. Due to insulin ineffectiveness, blood glucose levels in diabetic persons stay high. Consequently, inhibiting α-amylase and α-glucosidase enzymes is crucial for regulating glucose concentrations. Presently, several medications are available to block the α-amylase and α glucosidase enzymes, such as miglitol, acarbose, and voglibose, although with some adverse side effects. This work examines the synthesis of CuO-NPs as an alternative. The inhibition (%) of the enzymes α amylase Fig. 8A and α glucosidase Fig. 8B by CuO-NPs. The reduction in activity proportion for α-amylase varied from 91.7% at 1 mg/ml to 34.1% at 0.0019 mg/ml, while for α-glucosidase, it varied from 81.8% at 1 mg/ml to 32.3% at 0.0019 mg/ml. Prior studies demonstrated CuO-NPs’ capacity to inhibit α-amylase, and the inhibition manner goes along with our results^68,71–73^.

### Antiviral activity

**Fig. 9:**
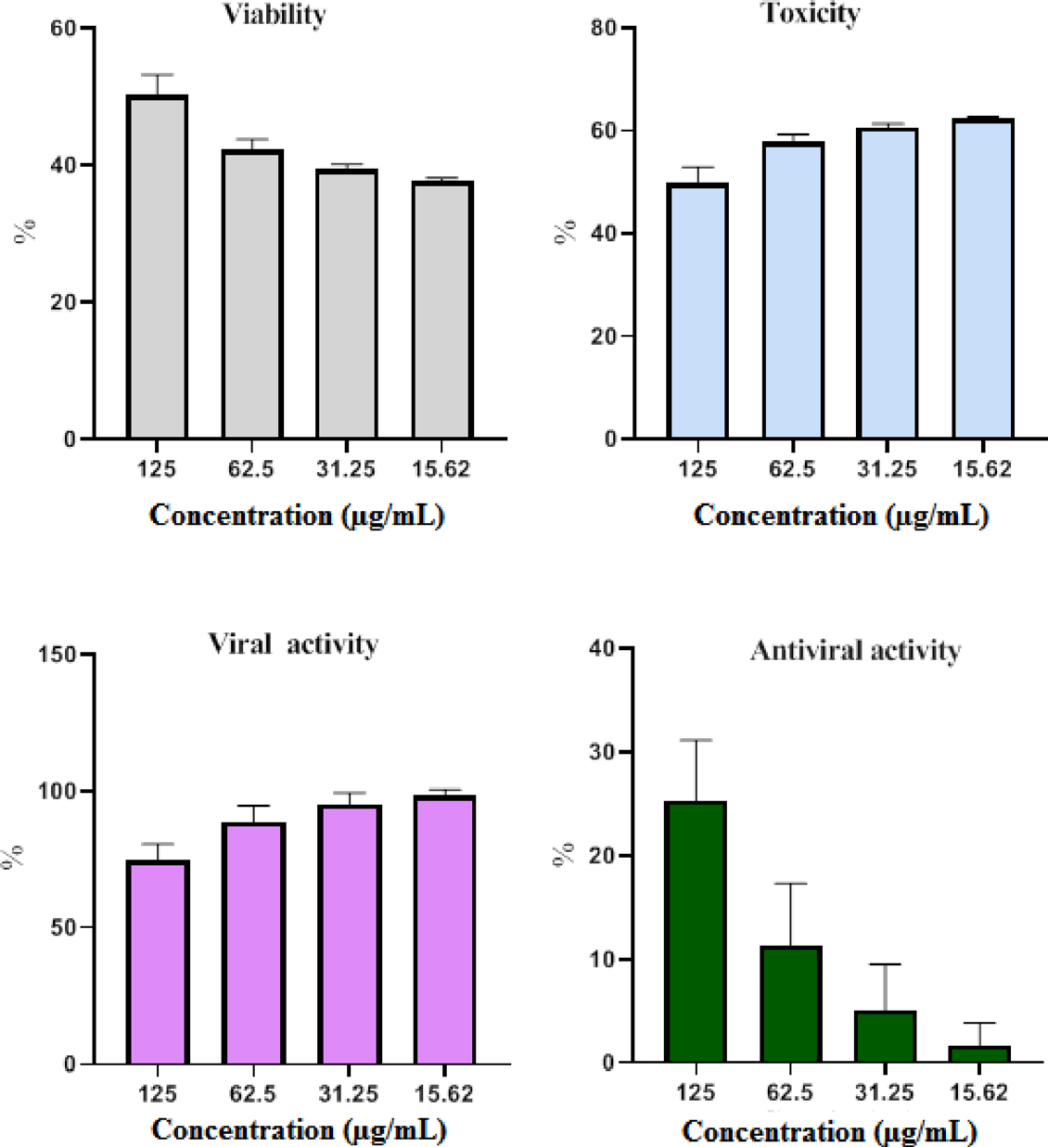
The effect of biofabricated CuO-NPs on HAV.

**Fig. 10:**
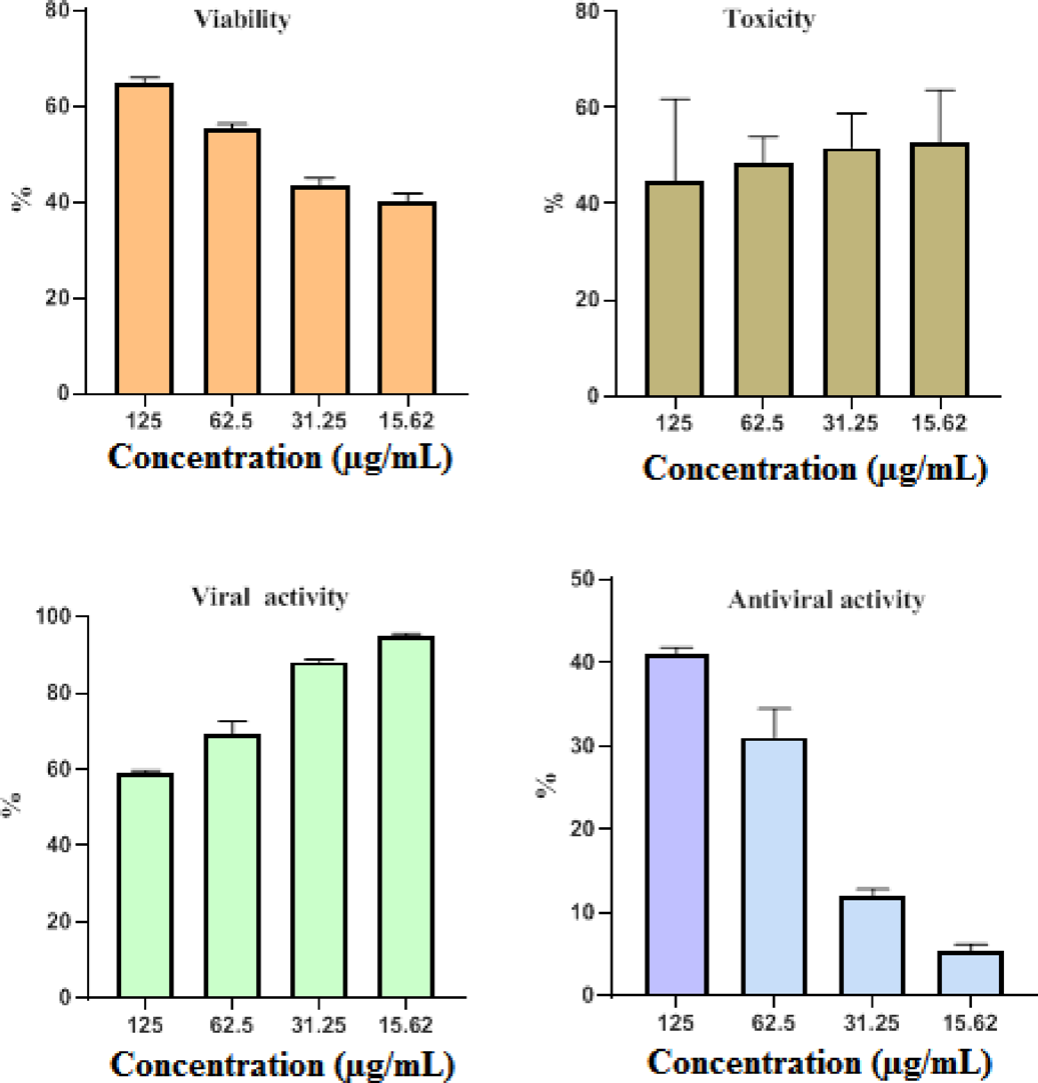
The effect of biofabricated CuO-NPs on CoXB4 virus.

This research examined the efficacy of CuO-NPs in reducing the growth of HAV and CoxB4 viruses. To determine the MNTC, CuOـNPs were assayed for cytotoxicity versus cell lines at 125 µg/mL. CuO-NPs were reported to exhibit promising antiviral properties against both HAV and CoxB4. At the same dose, COX B4 was more active than HAV. CuO-NPs showed 41.1% antiviral activity against COXB4 and 25.2% effectiveness against HAV at 125 µg/mL (Figs. 9 and 10 ). CuO-NPs shown remarkable antiviral efficacy versus both HAV and COXـB4, showing their potential for usage in biological areas. The antiviral efficacy of nanoparticles (NPs) has been elucidated through various mechanisms, which includes the inhibition of viral and cellular reactions that obstruct infection, connections between nano and specific cell-surfaces or receptors that impede viral entry, suppression of viral propagation, avoidance of viral dissemination, augmentation of oxidative stress through ROS generation, induction of cellular-apoptosis, and improvement of the host cell’s defenses against infection^74–76^. The decrease in virus frequency after administration with CuO-NPs underscores the pivotal function of copper, suggesting that copper nanoparticles provide significant potential for the development of potent antiviral therapies^77,78^. Copper is effective versus immuno-deficiency virus (HIV-1), as well as SARS-CoV-2. Direct 1 h interaction with SARS-CoV-2 onto surfaces coated in Cu NPs resulted in a considerable reduction of the virus’s titer (antiviral agents). The antiviral action of Cu included the destruction of virions by oxidation of virus capsid proteins, impairment with respect to their envelope, and denatured state of viral nucleic acids^79^. CuO-NPs might be useful in inactivating virus H1N1-influenza. Bronchial and renal cells infection with H1N1 and stimulated with 100 nm spherical CuO-NPs exhibited increased resistance to the cytopathic consequences of the virus. In the course of viral production, a markedly reduced amount of influenza virus nucleoproteins was observed after a 30 min exposure to Cu NPs. This validates the relevance of Cu NPs in the protection of virus-associated infectious illnesses^25,80^.

## Conclusion

The unique technology characterisation of CuO-NPs in this study demonstrates its remarkable potential as multifunctional bioactive materials with a variety of eco-friendly properties. CuO-NPs biosynthesis from *Opuntia ficus-indica* extract produces encouraging application outcomes, demonstrating exceptional effectiveness across a range of tests. Characterize of CuO-NPs was done via Uv, FTIR, XRD, SEM-EDX, DL and TEM. The antimicrobial effectiveness of CuO-NPs was performed against bacterial and fungal strain and MIC between 62.5 and 500 µg/mL. CuO-NPs shown an antioxidant activity via DPPH method with an IC_50_ of 165.5 µg/ml. Moreover, CuO-NPs demonstrated antibiofilm advantage versus MRSA and *P. aeruginosa*. The inhibitory effect of CuO-NPs towards bacterial strains may be caused by beta-1,3-glucanase, which interacts hydrophobically with amino acid residues in the active site, according to a molecular docking modelling. Furthermore, CuO-NPs displayed a noteworthy antiviral property against COXB4 and HAV at a dosage of 125 µg/mL, with antiviral efficacy of 40.9% and 28.6%, respectively. Additionally, 91.5% suppression of α-amylase and 82.3% inhibition of α-glucosidase were demonstrated by the CuO-NPs at 1000 µg/mL, confirming their antidiabetic qualities. CuO-NPs therefore hold great potential as an anti-inflammatory medication. The development of CuO-NPs in clinical and commercial applications is based on these discoveries, which validate its potential in antibacterial, antibiofilm, antiviral, anticancer, and antioxidant treatments.

## Data Availability

The data used to support the findings of this study are available from the corresponding author upon request.
